# LMO3 promotes proliferation and metastasis of papillary thyroid carcinoma cells by regulating LIMK1-mediated cofilin and the β-catenin pathway

**DOI:** 10.1515/med-2022-0419

**Published:** 2022-03-07

**Authors:** Zeyi Ling, Xiaoli Long, Ying Wu, Jie Li, Mingliang Feng

**Affiliations:** Department of Otorhinolaryngology Head and Neck Surgery, Yongchuan Hospital of Chongqing Medical University, Chongqing, 402160, China; Department of Geriatrics, Yongchuan Hospital of Chongqing Medical University, No. 439, Xuanhua Road, Yongchuan District, Chongqing, 402160, China

**Keywords:** LMO3, papillary thyroid carcinoma, metastasis, proliferation, LIMK1, cofilin, β-catenin

## Abstract

LIM domain only 3 (LMO3) interacts with transcription factors to regulate target genes involved in embryonic development. The oncogenic role of LMO3 in hepatocellular carcinoma, gastric cancer, and neuroblastoma has been reported recently. However, little is known about the biological function of LMO3 in papillary thyroid carcinoma (PTC). First, expression of LMO3 was dramatically enhanced in the PTC tissues and cell lines. Second, knockdown of LMO3 in PTC cells repressed cell proliferation and promoted cell apoptosis with downregulated Bcl-2 and upregulated cleaved caspase-3/PARP. *In vitro* cell migration and invasion of PTC were also retarded by siRNA-mediated silence of LMO3. Third, protein expression of LIM kinase (LIMK) 1-mediated phosphorylation of cofilin and nuclear translocation of β-catenin were reduced by the knockdown of LMO3. pcDNA-mediated overexpression of LIMK1 promoted cofilin phosphorylation and attenuated LMO3 silence-induced decrease of cofilin phosphorylation. Last, enhanced LIMK1 expression promoted PTC cell proliferation and metastasis and counteracted the suppressive effects of LMO3 silence on PTC cell proliferation and metastasis. In conclusion, LMO3 promoted PTC cell proliferation and metastasis by regulating LIMK1-mediated cofilin and the β-catenin pathway.

## 1 Introduction

Thyroid cancer, including papillary thyroid carcinoma (PTC), follicular thyroid cancer (FTC), medullary thyroid cancer (MTC), and anaplastic thyroid cancer (ATC), is the most common cancer of the endocrine system [1]. The increased incidence and the stable mortality rate of thyroid cancer appear to be due to the devoid of effective therapeutic strategies [1]. PTC, the most common subtype of thyroid cancer, accounts for 80% of all the cases [2]. PTC presents polycentrality in the thyroid gland and often metastases to local lymph nodes, which increases morbidity and mortality [3]. Therefore, it is necessary to find effective diagnostic markers or therapeutic targets for the treatment of PTC.

LIM domain only 3 (LMO3) belongs to the LMO protein family and is involved in the differentiation of various cells and the development of embryos, thus playing an important role in the development of the nervous system [4]. Moreover, LMO3 has been reported to regulate tumor signal transduction pathways through binding with other transcriptional factors. For example, LMO3 is a key downstream target of transcription signal of and participates in the NK2 Homeobox 1-mediated occurrence of lung cancer [5]. Nescient helix-loop-helix 2 binds to LMO3 to downregulate the expression of hes family bHLH transcription factor 1 through transactivation of achaete-scute complex-like 1 and induces malignant transformation of neuroblastoma [6]. LMO3 also binds to the tumor suppressor gene p53 and inhibits the transcriptional activation of apoptotic proteins downstream of p53 [7]. Therefore, LMO3 was considered to be an oncogene in the progression of gastric cancer [8], glioma [9], and hepatocellular carcinoma [10]. Recent research has reported that the expression level of LMO3 was increased in thyroid tumor [11]. However, little is known about the biological function of LMO3 in tumorigenicity of PTC.

Research has shown that LMO3 directly interacts with large tumor suppressor kinase (LATS) 1 to inhibit the phosphorylation of LATS1 and promote Rho GTPases activities, thus suppressing Hippo signal to promote the invasion and metastasis of hepatocellular carcinoma [10]. LATS1 binds to LIM kinase (LIMK) 1 and inhibits the activity of LIMK1 to regulate cytokinesis [12], and LIMK1 was implicated in the pathogenesis of thyroid cancer [13] and ATC [14]. Therefore, we hypothesized that LMO3 might affect the activity of LIMK1 to participate in the tumorigenicity of PTC.

In this study, the expression pattern and role of LMO3 in PTC were examined, and the mechanism of LMO3-mediated PTC cell metastasis was investigated by the loss of functional assays.

## 2 Materials and methods

### 2.1 Human tumor tissues

Pairs of PTC and adjacent normal tissues (*N* = 43) were acquired from patients that were recruited at the Yongchuan Hospital of Chongqing Medical University from 2015 to 2019 through thyroidectomies. All the patients signed informed consent. This study was approved by Yongchuan Hospital of Chongqing Medical University and in accordance with the 1964 Helsinki Declaration and its later amendments for ethical research involving human subjects.

### 2.2 Immunohistochemistry

PTC and adjacent normal tissues were fixed with 10% formalin and then embedded in paraffin. Formalin-fixed and paraffin-embedded tissues were then sectioned into 4 µm thick sections. The sections were incubated with 3% H_2_O_2_, and then immersed in Tris-EDTA buffer (pH 9.0) with 0.05% Tween 20 for 30 min at 95°C. After blocking in 4% dry milk and 0.3% goat serum, the sections were incubated overnight with anti-LMO3 antibody (1:100; Abcam, Cambridge, MA, USA). Following incubation with horseradish peroxidase-labeled secondary antibody and counterstaining with hematoxylin, the slides were examined under a light microscope (Olympus, Tokyo, Japan).

### 2.3 Cell culture

Human PTC cell lines (TPC-1, CAL62, IHH-4) and normal human thyroid cell lines (Nthy-ori3-1) were purchased from Shanghai Huiying biological technology (Shanghai, China). Cells were cultured in RPMI-1640 medium containing 10% fetal bovine serum (Lonza, Basel, Switzerland) in a 37°C incubator.

### 2.4 Cell transfection

siRNAs targeting LMO3 (siLMO3-1#: F: 5′-GGACUACGAGGAAGGUUUAdTdT-3′, R: 5′-UAAACCUUCCUCGUAGUCCdTdT-3′; or 2#: F: 5′-GCUGCAACCGAAAGAUCAAdTdT-3′, R: 5′-UUGAUCUUUCGGUUGCAGCdTdT-3′) and the negative control (siNC: 5′-CCAUUCCGAUCCUGAUCCG-3′) were synthesized by GenePharma (Suzhou, China). pcDNA vector was used to upregulate LIMK1. TPC-1 and CAL62 were seeded into 96-well plates and transfected with siNC, siLMO3-1#, or 2# via Lipofectamine 2000 (Invitrogen, Carlsbad, CA, USA). TPC-1 and CAL62 were also cotransfected with siLMO3-2# and pcDNA-LIMK1 by Lipofectamine 2000. Two days later, the cells were conducted with functional assays.

### 2.5 qRT-PCR

The transfected TPC-1 and CAL62 were performed with RNAiso Plus reagent (Takara, Kusatsu, Japan) for the isolation of RNAs. RNA was then reverse-transcribed into cDNA with the First Strand cDNA Synthesis Kit (Thermo Fisher Scientific, Waltham, MA, USA), and the qRT-PCR analysis was performed with Power SYBR Green PCR Master Mix (Applied Biosystems, Foster City, CA, USA). The primer sequences of selected genes are listed in Table 1 with GAPDH as the endogenous control. The fold change of SFRP1 was calculated with 2^−ΔΔCt^ with the following primers.

**Table 1:** Primers

ID	Sequence (5′-3′)
GAPDH F	AGGTCGGTGTGAACGGATTTG
GAPDH R	TGTAGACCATGTAGTTGAGGTC
LMO3 F	TCTGAGGCTCTTTGGTGTAACG
LMO3 R	CCAGGTGGTAAACATTGTCCTTGx

### 2.6 Cell viability and EdU staining

The transfected TPC-1 and CAL62 were reseeded into the 96-well plate and incubated for 24, 48, and 72 h. A total of 10 µL of CCK-8 solution (Beyotime, Beijing, China) was added to each well and incubated for 1 h. A Microplate Reader (BioTek, Winooski, VT, USA) was then used to measure the absorbance at 450 nm. For EdU staining, the transfected TPC-1 and CAL62 were reseeded into the 96-well plate and incubated with 50 µM EdU (RiboBio, Guangzhou, China) for 12 h. Paraformaldehyde-fixed cells were incubated with 2 mg/mL glycine and then with 0.5% Triton X-100. EdU antibody (1:500; Abcam) was used to stain the cells, and DAPI was used to stain the nuclei. The Apollo staining reaction buffer was used for EdU immunostaining, and the cells were examined under a microscope (Olympus).

### 2.7 Flow cytometer

The transfected TPC-1 and CAL62 were detached by trypsinization and then resuspended in the binding buffer of Annexin V-FITC/PI double staining apoptosis detection kit (KeyGEN BioTech, Jiangning, Nanjing, China). Cells were then stained with 5 µL of PI and 5 µL of annexin V (KeyGEN BioTech). The apoptotic ratio was analyzed with a FACS flow cytometer (Life Technologies, Darmstadt, Germany).

### 2.8 Western blot

The transfected TPC-1 and CAL62 were lysed with RIPA buffer (Ding Guo Chang Sheng Biotech, Beijing, China) for 30 min on ice. Following centrifugation at 12,000×*g*, the concentrations of the cell lysates were measured with a Pierce BCA Protein Assay Kit (Thermo Fisher Scientific). The cell lysates (30 µg) were analyzed with SDS-PAGE, and electro-transferred onto the PVDF membrane (Thermo Fisher Scientific). The primary antibodies, including anti-LATS1 (ab70561) and anti-p-LATS1 (ab111344) (1:1,500; Abcam); anti-YAP (ab52771) and anti-p-YAP (ab62751) (1:2,000; Abcam); anti-LMO3 (ab230490), anti-LIMK1 (ab39641), and anti-p-LIMK1 (ab194798) (1:2,500; Abcam); anti-Bcl-2 (ab194583) and anti-β-actin (ab8227) (1:3,000; Abcam); anti-cleaved caspase-3 (ab2302) and anti-cleaved PARP (ab4830) (1:3,500; Abcam); anti-cofilin (ab42824) and anti-p-cofilin (ab12866) (1:4,000; Abcam); anti-β-catenin (ab6302), anti-β-tubulin (ab6046), and anti-Histone H3 (ab18521) (1:4,500; Abcam); were used to probe the membranes that were blocked with 5% bovine serum albumin. The membranes were incubated with horseradish peroxidase-conjugated immunoglobulin G (ab6721) (1:6,000; Abcam), and the blots were detected by enhanced chemiluminescence (KeyGen, Nanjin, China).

### 2.9 Wound healing and transwell assays

The transfected TPC-1 and CAL62 were reseeded into 6-well plates for 24 h. A sterile pipette tip was used to generate a scratch. The widths of the scratches were calculated 48 h later under a microscope. For transwell assay, the transfected TPC-1 and CAL62 in serum-free medium were planted in the apical chamber with Matrigel (Biosciences, San Jose, CA, USA). The medium containing 20% fetal bovine serum was added into the basolateral chamber. After 24 h, cells in the basolateral chamber were fixed in 10% formaldehyde, stained with 0.1% crystal violet, and measured under a microscope.

### 2.10 Statistical analysis

Results of the experiments performed in triplicates independently were presented as mean ± SD. Statistical analyses between different groups were performed with one-way analysis of variance or Student’s *t*-test with SPSS19.0 software. Values were considered significant at *p* < 0.05.


**Ethics approval:** Ethical approval was obtained from the Ethics Committee of thw Yongchuan Hospital of Chongqing Medical University.
**Statement of informed consent:** Written informed consent was obtained from a legally authorized representative(s) for anonymized patient information to be published in this article.

## 3 Results

### 3.1 Upregulation of LMO3 in PTC

**Figure 1:**
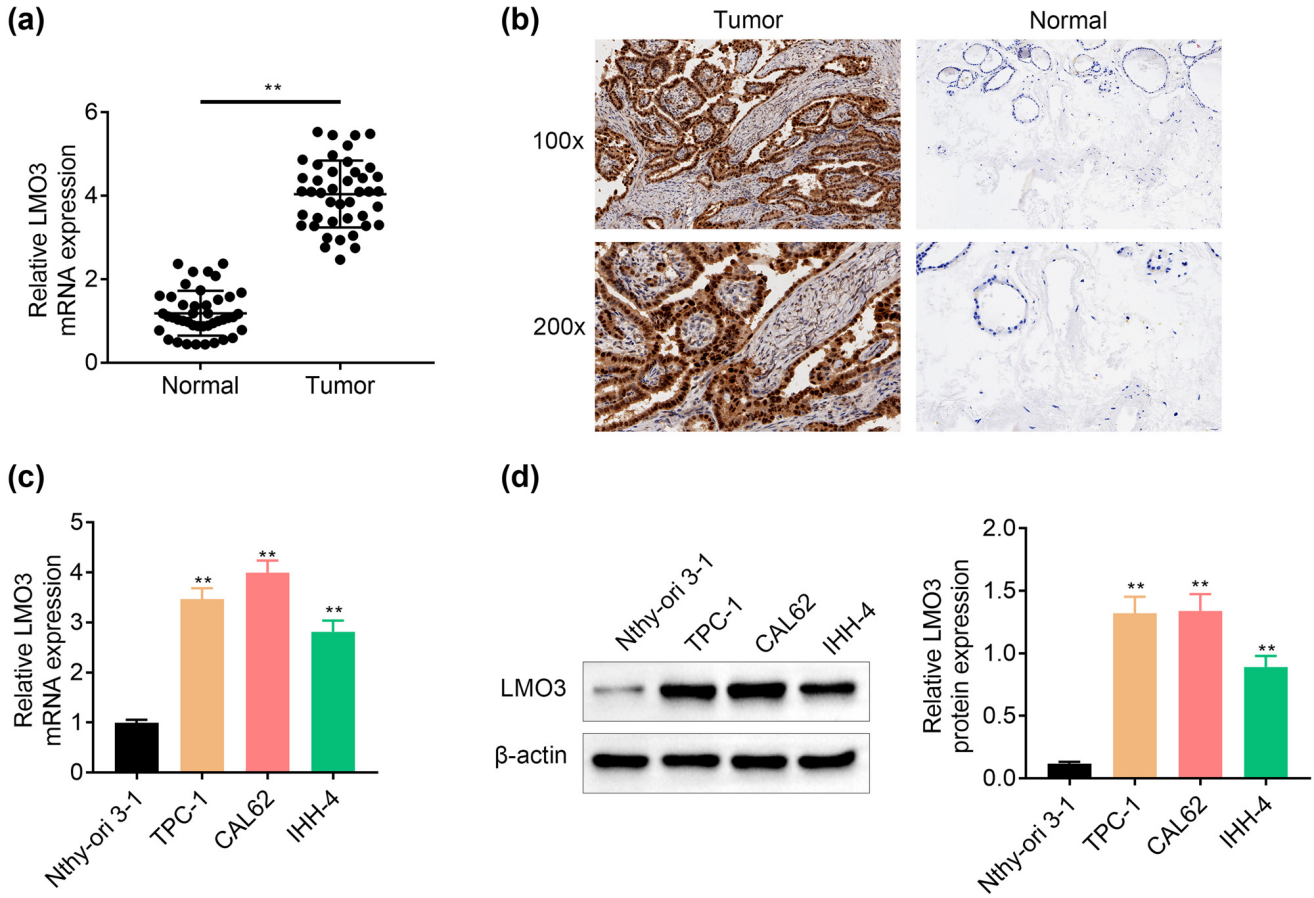
Upregulation of LMO3 in PTC. (a) LMO3 expression was elevated in the PTC tissues compared to that in the adjacent normal tissues via qRT-PCR analysis. (b) LMO3 expression was elevated in the PTC tissues compared to that in the adjacent normal tissues via immunohistochemical analysis. (c) LMO3 expression was elevated in the PTC cell lines (TPC-1, CAL62, IHH-4) compared to that in the normal human thyroid cell line (Nthy-ori3-1) via qRT-PCR analysis. (d) LMO3 expression was elevated in the PTC cell lines (TPC-1, CAL62, IHH-4) compared to that in the normal human thyroid cell line (Nthy-ori3-1) via western blot analysis. ** vs normal or Nthy-ori3-1, *p* < 0.01.

We first measured mRNA expression of LMO3 in 43 pairs of PTC and adjacent normal tissues by qRT-PCR. The result showed that LMO3 expression was elevated in the PTC tissues compared to the adjacent normal tissues (Figure 1a). Immunohistochemical analysis also confirmed the higher expression of LMO3 in the PTC tissues than the adjacent normal tissues (Figure 1b). Moreover, we identified the higher mRNA (Figure 1c) and protein (Figure 1d) expression of LMO3 in the human PTC cell lines (TPC-1, CAL62, IHH-4) than that in the normal human thyroid cell line (Nthy-ori3-1), suggesting the possible relation between LMO3 and PTC progression.

### 3.2 LMO3 promoted PTC cell proliferation

**Figure 2:**
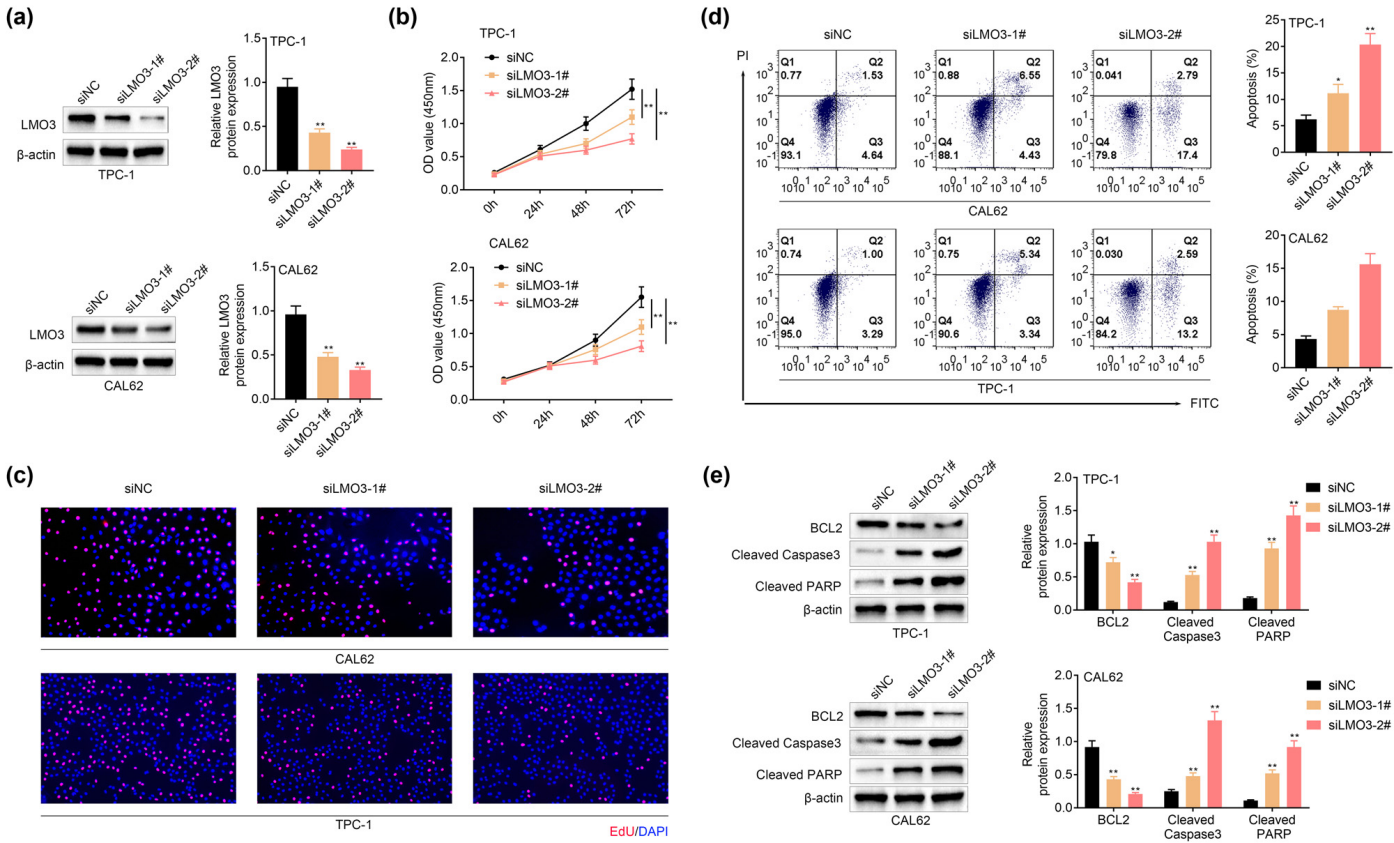
LMO3 promoted PTC cell proliferation. (a) LMO3 protein expression was downregulated in the PTC cell lines (TPC-1, CAL62) by transfection with siLMO3-1# and 2#. Knockdown of LMO3 (b) decreased cell viability of TPC-1 and CAL62, (c) reduced the cell proliferation of TPC-1 and CAL62, (d) promoted the cell apoptosis of TPC-1 and CAL62, and (e) enhanced protein expression of cleaved caspase-3 and cleaved PARP and reduced Bcl-2 in TPC-1 and CAL62. ** vs siNC, *p* < 0.01.

To unravel the regulatory role of LMO3 on PTC progression, TPC-1 and CAL62 were transfected with siRNAs targeting LMO3. Western blot analysis showed lower expression of LMO3 by siLMO3-1# and 2# (Figure 2a). Knockdown of LMO3 decreased cell viability of TPC-1 and CAL62 (Figure 2b), reduced cell proliferation (Figure 2c), and promoted cell apoptosis (Figure 2d). Protein expression of cleaved caspase-3 and cleaved PARP were enhanced, while Bcl-2 was reduced in TPC-1 and CAL62 that were transfected with siLMO3-1# and 2# (Figure 2e), suggesting the anti-proliferative and pro-apoptotic effects of LMO3 silence on PTC.

### 3.3 LMO3 promoted PTC cell metastasis

**Figure 3:**
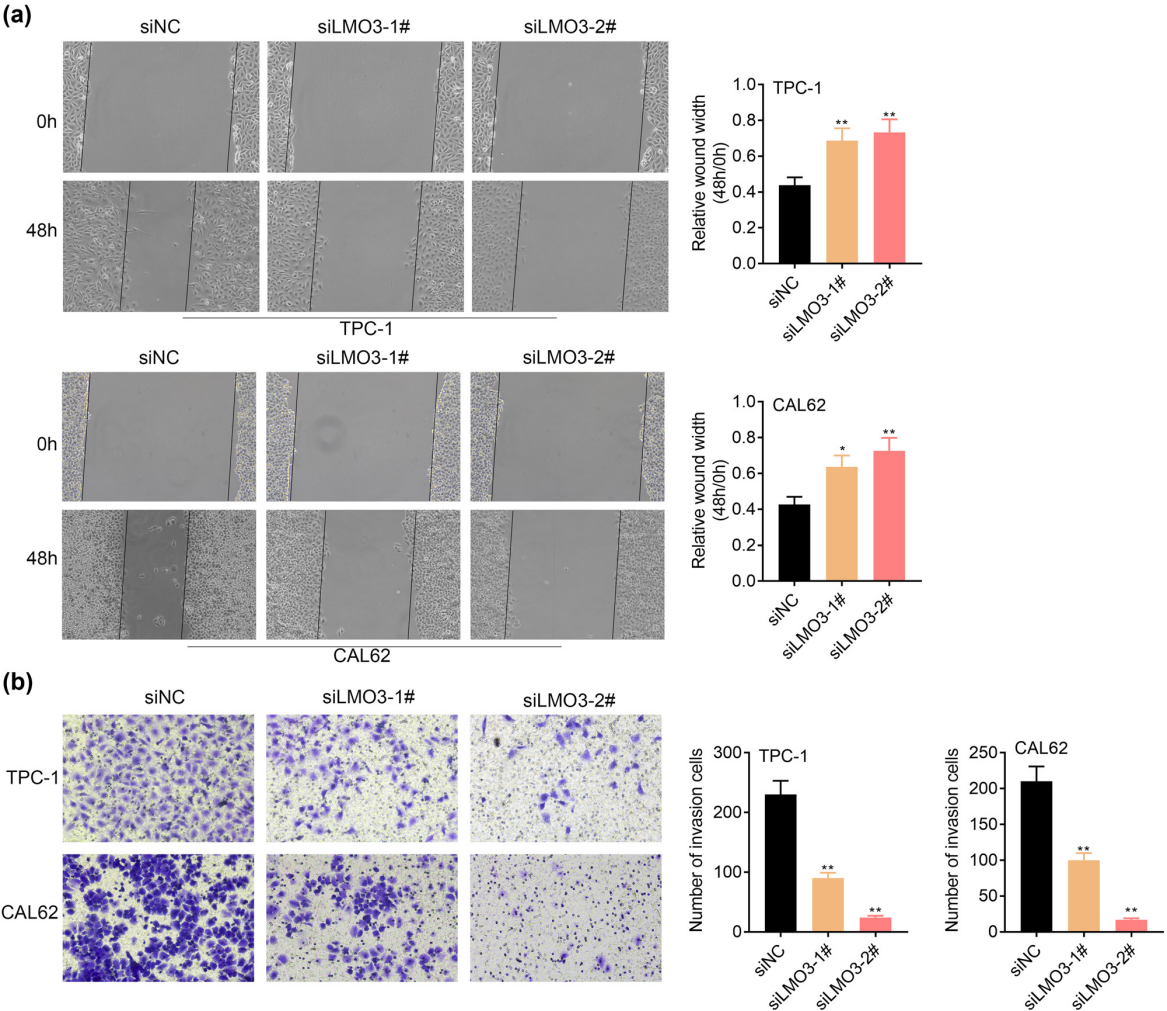
LMO3 promoted PTC cell metastasis. Knockdown of LMO3 (a) repressed cell migration of TPC-1 and CAL62 and (b) repressed cell invasion of TPC-1 and CAL62. ** vs siNC, *p* < 0.01.

Cell migrations of TPC-1 and CAL62 were suppressed by the knockdown of LMO3 (Figure 3a). Moreover, transfection with siLMO3-1# and 2# repressed the cell invasion of TPC-1 and CAL62 (Figure 3b). These results demonstrated the anti-invasive effect of LMO3 silence on PTC.

### 3.4 LMO3 contributed to LIMK1-mediated activation of cofilin and β-catenin pathways

**Figure 4:**
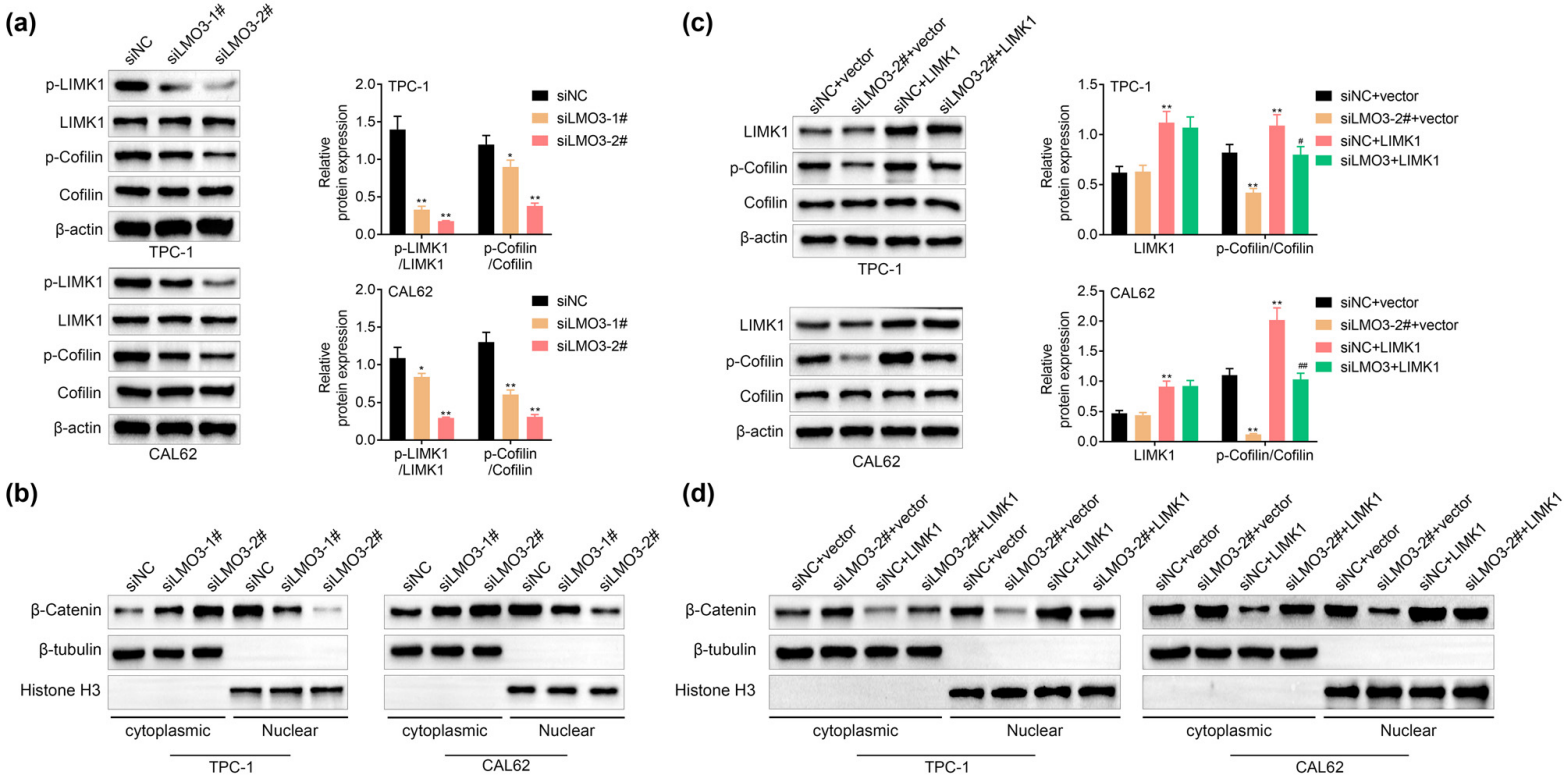
LMO3 contributed to LIMK1-mediated activation of cofilin and β-catenin pathways. (a) Knockdown of LMO3 decreased protein expression of cofilin and LIMK1 phosphorylation, while it had no significant effect on LIMK1 and cofilin in TPC-1 and CAL62. (b) Knockdown of LMO3 increased protein expression of cytoplasmic β-catenin and decreased nuclear translocation of β-catenin in TPC-1 and CAL62. (c) Overexpression of LIMK1 promoted protein expression of cofilin phosphorylation and attenuated LMO3 silence-induced decrease of cofilin phosphorylation in TPC-1 and CAL62. (d) Overexpression of LIMK1 attenuated LMO3 silence-induced upregulation of cytoplasmic β-catenin and downregulation of nuclear β-catenin in TPC-1 and CAL62. ** vs siNC or siNC + vector, *p* < 0.01. #, ## vs siNC + LIMK1, *p* < 0.05, *p* < 0.01.

**Figure A1:**
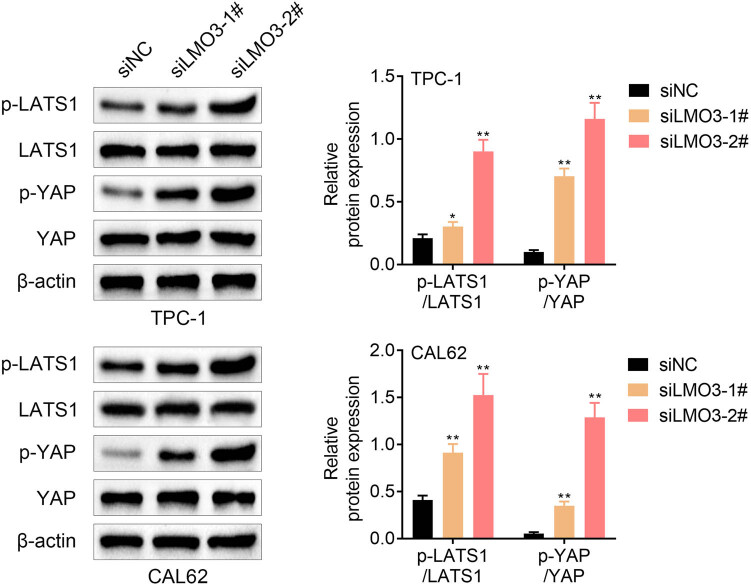
Knockdwon of LMO3 reduced the phosphorylation of LATS1 and YAP expression in TPC-1 and CAL62. *, ** vs siNC, *p* < 0.05, *p* < 0.01.

Protein expressions of LIMK1 and cofilin were not affected by the knockdown of LMO3 in TPC-1 and CAL62 (Figure 4a). However, phosphorylation of LIMK1 and cofilin was reduced by knockdown of LMO3 (Figure 4a). Moreover, protein expression of cytoplasmic β-catenin was enhanced, while nuclear β-catenin was reduced in TPC-1 and CAL62 that were transfected with siLMO3-1# and 2# (Figure 4b), while the nuclear translocation of β-catenin was decreased by knockdown of LMO3 (Figure 4b). Overexpression of LIMK1 promoted phosphorylation of cofilin (Figure 4c) and attenuated LMO3 silence-induced decrease of cofilin phosphorylation (Figure 4c). Moreover, overexpression of LIMK1 attenuated LMO3 silence-induced upregulation of cytoplasmic β-catenin and downregulation of nuclear β-catenin (Figure 4d), suggesting that silence of LMO3 suppressed LIMK1-mediated activation of cofilin and β-catenin in PTC. The knockdown of LMO3 reduced the phosphorylation of LATS1 and YAP in TPC-1 and CAL62 (Figure A1), thus promoting the activation of the Hippo signaling pathway in PTC.

### 3.5 Overexpression of LIMK1 counteracted with the suppressive effects of LMO3 silence on the PTC cell growth and metastasis

**Figure 5:**
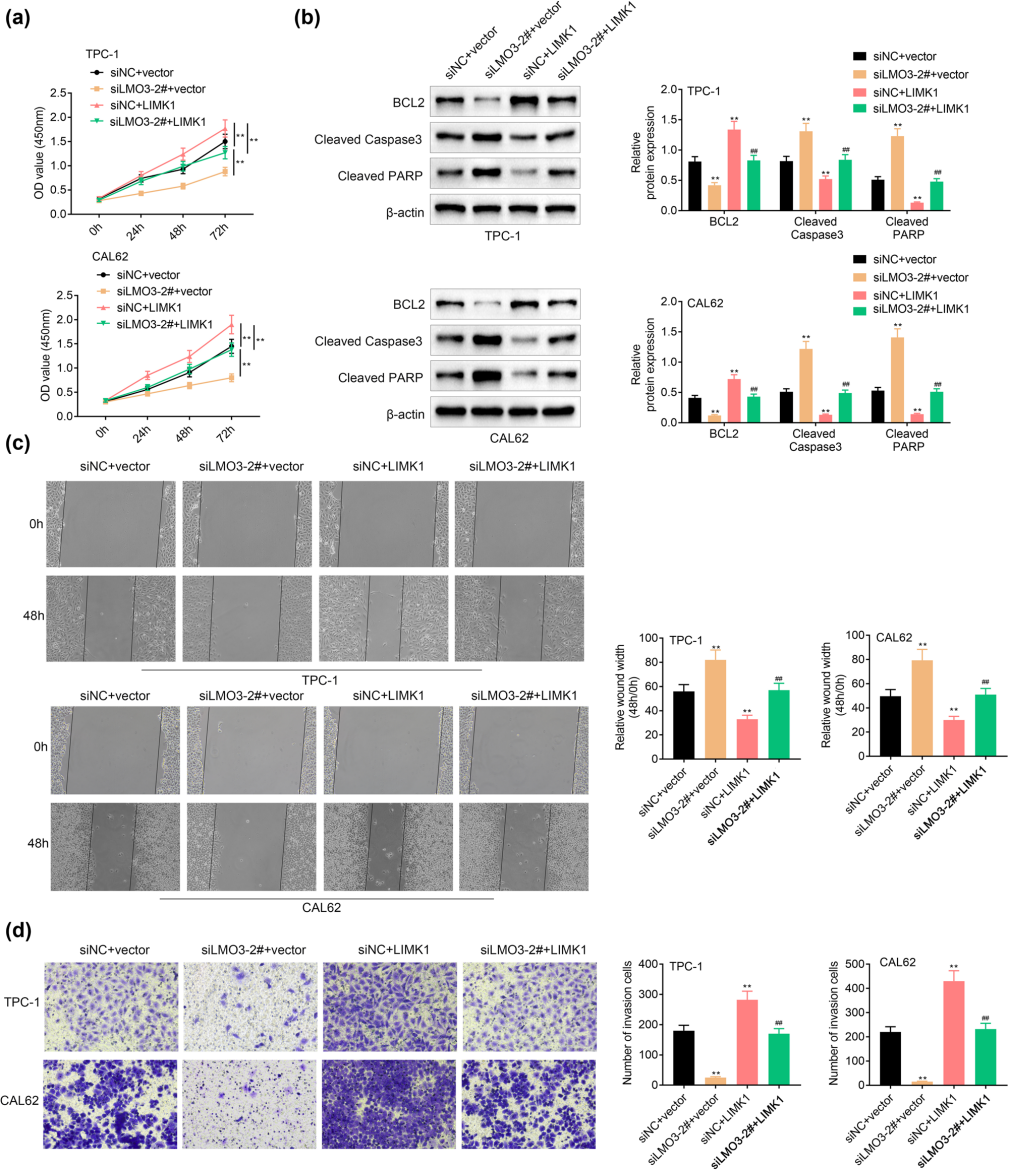
Overexpression of LIMK1 counteracted the suppressive effects of LMO3 silence on PTC cell growth and metastasis. (a) Overexpression of LIMK1 increased cell viability of TPC-1 and CAL62 and weakened silence of LMO3-induced decrease of PTC cell viability. (b) Overexpression of LIMK1 increased protein expression of Bcl-2, decreased cleaved caspase-3 and cleaved PARP in TPC-1 and CAL62, and weakened silence of LMO3-induced decrease of Bcl-2 and increase of cleaved caspase-3 and cleaved PARP. (c) Overexpression of LIMK1 promoted cell migration of TPC-1 and CAL62 and weakened silence of LMO3-induced decrease of PTC cell migration. (d) Overexpression of LIMK1 promoted cell invasion of TPC-1 and CAL62 and weakened silence of LMO3-induced decrease of PTC cell invasion. ** vs siNC or siNC + vector, *p* < 0.01. ## vs siNC + LIMK1, *p* < 0.01.

TPC-1 and CAL62 were cotransfected with siLMO3-2# and pcDNA-LIMK1 to investigate the role of the LMO3/LIMK1 axis on PTC progression. Overexpression of LIMK1 increased the cell viability of TPC-1 and CAL62 (Figure 5a) and weakened the silence of LMO3-induced decrease of PTC cell viability (Figure 5a). Overexpression of LIMK1 showed reversed effects on protein expression of Bcl-2, cleaved caspase-3, and cleaved PARP compared to the silence of LMO3 (Figure 5b), and ectopic LIMK1 expression attenuated LMO3 silence-induced decrease of Bcl-2 and increase of cleaved caspase-3 and cleaved PARP (Figure 5b). Moreover, LIMK1 overexpression promoted cell migration (Figure 5c) and invasion (Figure 5d) of TPC-1 and CAL62, and counteracted the suppressive effects of LMO3 silence on PTC cell metastasis.

## 4 Discussion

The LMO protein family contains conserved LIM domains to bind with other transcriptional factors to mediate gene expression programs during developmental processes, thus participating in the onset and progression of cancers, such as neuroblastoma, breast cancer, and T cell leukemia [15]. Activation of LMO4 expression was implicated in the progression of PTC [16]. Since LMO3 was found to be upregulated in the thyroid tumor [11], the biological function of LMO3 on PTC progression was investigated in this study.

Our results first demonstrated that LMO3 was elevated in PTC tissues and cells. The previous study has shown that LMO3 expression was significantly associated with disease-free survival and overall survival of patients with gastric cancer [8], and the relation between LMO3 expression and clinicopathological factors of PTC patients should be investigated to suggest the diagnostic or prognostic roles of LMO3 on PTC. The oncogenic role of LMO3 on PTC was then identified, and it demonstrated that knockdown of LMO3 reduced cell viability of PTC, promoted cell apoptosis, and suppressed cell proliferation, migration, and invasion.

LATS1, the key regulator of the Hippo pathway, was found to be a binding partner of LMO3 during tumorigenesis of hepatocellular carcinoma [10]. Upregulation of LATS1 through downregulation of miR-103a-3p repressed malignancy of thyroid cancer [17]. Moreover, LATS1 colocalizes with LIMK1 to regulate cytokinesis [12] and mediates activation of LIMK1 through phosphorylation of kinesin-like motor protein KIF23 [18]. LIMK1 phosphorylates the potent regulator of actin filament dynamics, cofilin, to regulate cell migration [19] and actin dynamics [20]. Here, our results showed that knockdown of LMO3 decreased protein expression of cofilin phosphorylation, while it had no significant effects on LIMK1 and cofilin expression. Moreover, overexpression of LIMK1 promoted cofilin phosphorylation and attenuated the silence of LMO3-induced decrease of cofilin phosphorylation. The phosphorylation of LIMK1 should be investigated to unravel whether LMO3 contributed to LIMK1 phosphorylation to promote cofilin phosphorylation.

A previous study has shown that LIMK1-mediated phosphorylation of cofilin promoted colorectal cancer progression [21], and silence of LIMK1/cofilin pathway abrogated tumor cell growth and metastasis [22]. Moreover, the active form of cofilin was implicated in aurora kinase A-induced PTC metastasis [23]. Overexpression of LIMK1 in this study enhanced the cell viability of PTC, reduced cell apoptosis, and promoted migration and invasion. In addition, ectopic expression of LIMK1 attenuated knockdown of LMO3-induced inhibition of PTC cell growth and metastasis. Therefore, LMO3 might contribute to PTC progression through LIMK1-mediated cofilin phosphorylation.

LIMK1 was reported to be overexpressed in colorectal cancer tissues and functioned as a competitive inhibitor of LIMK2 to promote the nuclear translocation of β-catenin, thus promoting tumor progression through activation of the Wnt/β-catenin pathway [24]. Enhanced Wnt/β-catenin pathway was essential for the cell growth and survival of PTC [25]. Our results showed that knockdown of LMO3 decreased the nuclear translocation of β-catenin in PTC cells, suggesting that LMO3 might also contribute to PTC progression through LIMK1-mediated nuclear translocation of β-catenin.

In conclusion, this study for the first time proved that reduced LMO3 expression in PTC promoted apoptosis, and suppressed proliferation, migration, and invasion. LIMK1-mediated cofilin phosphorylation and β-catenin nuclear translocation were identified as novel mechanisms of LMO3 in tumors. These results would advance the understanding of the pathogenesis of PTC and might provide a potential therapeutic target for the treatment of PTC.
